# Usefulness of Lipid Apheresis in the Treatment of Familial Hypercholesterolemia

**DOI:** 10.1155/2014/864317

**Published:** 2014-10-19

**Authors:** Matthew Lui, Ross Garberich, Craig Strauss, Thomas Davin, Thomas Knickelbine

**Affiliations:** ^1^Minneapolis Heart Institute Foundation at Abbott Northwestern Hospital, 920 E 28th Street, Minneapolis, MN 55407, USA; ^2^Minneapolis Heart Institute at Abbott Northwestern Hospital, 920 E 28th Street, Minneapolis, MN 55407, USA; ^3^Kidney Specialists of Minnesota at Abbott Northwestern Hospital, 920 E 28th Street, Minneapolis, MN 55407, USA

## Abstract

Lipid apheresis is used to treat patients with severe hyperlipidemia by reducing low-density lipoprotein cholesterol (LDL-C). This study examines the effect of apheresis on the lipid panel and cardiac event rates before and after apheresis. An electronic health record screen of ambulatory patients identified 11 active patients undergoing lipid apheresis with 10/11 carrying a diagnosis of FH. Baseline demographics, pre- and postapheresis lipid levels, highest recorded LDL-C, cardiac events, current medications, and first apheresis treatment were recorded. Patients completed a questionnaire and self-reported risk factors and interest in alternative treatment. There were significant reductions in mean total cholesterol (−58.4%), LDL-C (−71.9%), triglycerides (−51%), high-density lipoprotein (HDL) cholesterol (−9.3%), and non-HDL (−68.2%) values. Thirty-four cardiac events were documented in 8 patients before apheresis, compared with 9 events in 5 patients after apheresis. Our survey showed a high prevalence of statin intolerance (64%), with the majority (90%) of participants indicating an interest in alternative treatment options. Our results have shown that lipid apheresis primary effect is a marked reduction in LDL-C cholesterol levels and may reduce the recurrence of cardiac events. Apheresis should be compared to the newer alternative treatment modalities in a randomized fashion due to patient interest in alternative options.

## 1. Introduction

Lipid apheresis provides a safe and effective means of treating patients with severe hyperlipidemia. It functions by first separating plasma from blood cells with a cell separator and then using either the adsorption of apolipoprotein B by affinity columns containing anti-apolipoprotein B antibodies or dextran sulphate, or their precipitation at low pH by heparin [1]. Lipid apheresis allows patients to attain lower levels of low-density lipoprotein (LDL), which are usually not attainable with traditional drug therapy alone, while leaving high-density lipoprotein (HDL) levels generally unaffected. When used in conjunction with statins and other lipid-lowering drugs, lipid apheresis may also induce the regression of coronary atherosclerotic plaque in familial hypercholesterolemia (FH) patients [2].

FH is a group of autosomal dominant genetic defects resulting in elevated serum (LDL) cholesterol levels. In the heterozygous state, FH is a relatively common but serious genetic disorder, with an incidence of about 1 in 500 persons in the general population. FH has been associated with an increased risk for atherosclerosis, premature coronary heart disease, and heart failure [3, 4]. FH is caused by a mutation affecting apolipoprotein B [5], proprotein convertase subtilisin kexin type 9 (PCSK9; an enzyme involved in LDL receptor degradation) [6], or, most commonly, the LDL receptor gene, resulting in defective LDL receptors and/or a diminished number of LDL receptors [7, 8]. These mutations cause LDL to be catabolized at a slower rate and thus accumulate in the circulation. Currently, FH is treated using a variety of cholesterol-lowering drugs, most notably statins or HMG-CoA reductase inhibitors. For many patients, however, statins are not a viable treatment option, because of either intolerance or ineffectiveness. Lipid apheresis is an alternative form of treatment for these FH patients as well as those who have persistently elevated LDL levels despite treatment.

Because apheresis is performed at only a few highly specialized centers in relatively low volume, there is very little literature discussing the effectiveness of lipid apheresis on the reduction of lipid profiles and the prevention of future cardiac events. This study, therefore, reports the experience in a single metropolitan center of treating patients with hyperlipidemia with lipid apheresis.

## 2. Methods

Retrospective chart reviews were performed and questionnaire surveys were given to active lipid apheresis patients at the Minneapolis Heart Institute (MHI) at Abbott Northwestern Hospital (ANW), Minneapolis, Minnesota. MHI and ANW are divisions of Allina Health, a large healthcare provider in Minnesota and western Wisconsin. Patients were identified through an electronic health record (EHR) screen of ambulatory patients representing all patients seen at all Allina Health metro area and regional locations between 2009 and 2012 (EPIC Systems, Verona WI). Of these patients, those currently undergoing lipid apheresis were identified and served as the study group. Criteria to qualify for apheresis were based on the United States Food and Drug Administration (FDA) approval recommendations. Currently, the FDA supports LDL apheresis for patients who, after six months, do not have an adequate response to diet therapy and maximum drug therapy, due to either ineffectiveness or intolerance, and meet the following criteria:

- functional homozygotes with an LDL cholesterol >500 mg/dL without CAD,
- functional heterozygotes with LDL cholesterol >300 mg/dL without CAD,
- functional heterozygotes with LDL cholesterol >200 mg/dL with documented coronary heart disease [9].

The date of birth, gender, date of apheresis initiation, lipid disorder diagnosis, apheresis frequency, and family history of cardiac events were recorded. Patients were noted as having FH if the active problem list contained a diagnosis of FH. To determine which patients had FH, we used the National Lipid Association (NLA) criteria for an 80% probable FH diagnosis, using the highest LDL recorded in the patient chart as follows: age <20 and LDL >190 mg/dL, age 20–29 and LDL >220 mg/dL, and age ≥30 and LDL >250 mg/dL [10]. Patients were also diagnosed by referring physicians as listed in the EHR. Potential homozygous FH (HoFH) patients were defined as having an untreated LDL >500 mg/dL or a treated (on statin) LDL over >300 mg/dL, in addition to clinical evidence of xanthomas before age of 10 years or having two parents with heart disease or high lipids. Identifiable secondary causes for marked hyperlipidemia were excluded from the analysis by examining the EHR chart of each potential homozygote.

Current cholesterol lowering medications were also recorded, focusing on the use of statins, colesevelam (Welchol), ezetimibe (Zetia), niacin, and aspirin. A significant cardiovascular event was defined as a myocardial infarction (MI), a percutaneous transluminal coronary angioplasty (PTCA) or stenting procedure, or a coronary artery bypass graft (CABG) using EHR documented ICD-9 criteria. Cardiac events were separated by their occurrence before or after the patient began apheresis, and the total number of events was recorded for each group. Multiple cardiac events occurring at the same hospitalization, such as MI followed by PTCA, were counted as a single event for cardiac event rate calculation. Pre- and postapheresis cardiac event rates were calculated by adding the total number of cardiac events and dividing by the total person years during each time period. The preapheresis time period describes the time from the first documented EHR visit to the date of apheresis initiation. The postapheresis time period describes the time from the date of apheresis initiation to the study date. Unverifiable events noted in the EHR but occurring prior to the first documented EHR visit were noted but excluded from the cardiac event rate calculation. Patients who started apheresis before EHR documentation were excluded from the preapheresis analysis. Mean acute LDL reductions were calculated by averaging all recorded LDL values prior to and immediately after the treatment sessions. Mean acute total cholesterol, HDL cholesterol, and triglyceride reductions were calculated by using lipid profile from the most recent treatment session.

LDL apheresis was performed at Abbott Northwestern Hospital using the Kaneka Liposorber LA-15 System (Kaneka Medical Products). The system consists of the Kaneka MA-03 machine, the integrated Sulflux KP-05 Plasma Separator, which consists of porous hollow fibers, to separate the plasma from the whole blood, and two disposable Liposorber LA-15 Adsorption columns to adsorb apolipoprotein B-containing lipoproteins from patient plasma. All patients underwent apheresis every two weeks.

*Questionnaire*. A phone questionnaire was given to all patients as shown in the appendix. Patients confirmed information in their EHR such as risk factors, answered questions relating to their awareness of FH and if their family had been previously tested for it, provided their level of satisfaction with their apheresis program, and indicated their interest in learning more about alternative treatments. The data from the questionnaires was cross-referenced with the data from the patient charts to ensure accuracy.

### 2.1. Statistical Analysis

Descriptive statistics are displayed as means and SDs for continuous variables; number and percentage with characteristic are given for categorical variables. Categorical variables were analyzed using Pearson's chi-square or Fisher's exact tests. Continuous variables were analyzed using Student's* t*-test. A value of *P* < 0.05 was considered significant, and *P* values are two-sided where possible. All statistical calculations and plots were done with Stata 11.2 (College Station, TX). Institutional Review Board approval was obtained for data collection, follow-up, and data analysis.

## 3. Results

### 3.1. Demographics

As of July 2013, there were 11 active participants in the apheresis program. Patient characteristics are shown in Table 1. Of these, 8 (72.7%) were male, 10 (90.9%) were Caucasian, 1 (9.1%) was African American, 10 (90.9%) carried the diagnosis of FH, with 2 (18.2%) patients identified as probable homozygotes, and 1 (9.1%) was diagnosed as having Familial Combined Hyperlipidemia. The average age of patients was 65.6 ± 9.3 years, and patients had been on apheresis for an average of 6.2 ± 7.0 years. Four (36.4%) patients were currently on statins while the other 7 (63.6%) had a history of statin intolerance. Five of 11 (45.5%) patients were on a nonstatin cholesterol lowering medications, including 1 (9.1%) on colesevelam (Welchol), 3 (27.3%) on ezetimibe (Zetia), and 1 (9.1%) on niacin. Nine of the 11 (81.8%) were on aspirin.

### 3.2. Lipid Profile Results

Average lipid profiles immediately before and immediately after apheresis are listed in Table 2. Maximum LDL levels ranged from 211 to 448 mg/dL with a mean (SD) value of 298 ± 80.7 mg/dL in the study group. Since our EHR was implemented in 2005, it is possible we may be underestimating the highest lifetime LDL for each patient.

### 3.3. Questionnaire Results

Results of the patient questionnaire are shown in Table 3. Of the 11 participants, 9 completed the questionnaire in its entirety; 1 patient provided answers to all questions but did not disclose risk factors and 1 patient did not complete the questionnaire. All of the patients indicated that they were aware that they likely had FH and 7 patients indicated that their immediate family had been tested for FH. All those surveyed indicated a family history of heart problems. The patients self-reported a total of 44 cardiac events before apheresis and 8 cardiac events after apheresis. Of the 10 patients that completed the questionnaire, 4 patients were currently on statins while the other 6 were statin intolerant. Nine patients were interested in learning about alternative treatments for FH.

### 3.4. Cardiac Events

Eight patients (72.7%) had a cardiac event documented by EHR, with 43 cardiac events occurring overall (Table 4). Self-reported events which were unable to be verified via the EHR were excluded from the cardiac event rate analysis. Thirty-four cardiac events were documented before apheresis in 8 patients compared with 9 events in 5 patients after apheresis. After excluding cardiac events that were unverifiable, 14 cardiac events were documented in the preapheresis time period and 7 were documented in the postapheresis time period. All postapheresis events occurred in patients with prior documented CAD. No de novo events occurred in prior asymptomatic patients. The cardiac event rates were calculated to be 0.23 (0.13, 0.39) events per person year in the preapheresis group and 0.10 (0.041, 0.21) events per person year in the postapheresis group (*P* = 0.064). Patients were observed for an average of 7.6 ± 5.9 years before apheresis and 6.2 ± 4.7 years after apheresis, with 60.6 total patient years before apheresis and 67.8 patient years after apheresis.

## 4. Discussion

This study was conducted to gain more information on lipid apheresis and evaluate the effectiveness in lowering lipid values. In addition, through chart review and patient survey, we attempted to gain a greater understanding of this patient population in terms of traditional risk factors, family awareness and screening, statin and other cholesterol medication uses, desire for additional treatment options, and ultimately cardiac events. Our study shows that apheresis markedly lowers total cholesterol, LDL cholesterol, triglycerides and, to a much lesser degree, HDL cholesterol. There was a small, but statistically significant, reduction in HDL values after apheresis.

Many of these patients were “statin intolerant” and some had been using nonstatin cholesterol medications. Importantly, 10/11 (90.9%) participants indicated a desire to learn more about other potential treatment options, indicating that this population may indeed experience fatigue of this procedure. Thus, there is a need for newer treatment options.

Although taken from a small study population, our data suggests a reduction in cardiac event rate after apheresis. While not statistically significant, our data shows a strong trend towards event rate reduction. This statistical insignificance is likely explained by the study's small sample size. With a larger population, the effects of lipid apheresis will become clearer. It is also important to note that the risk for cardiac events increases with age.

### 4.1. Comparison to Prior Studies

LDL apheresis has been shown to improve endothelium dependent vasodilation [11, 12], microvascular flow [13], and myocardial perfusion [14]. Some studies [2, 15, 16] have also shown a significant reduction in angiographic CAD, but others have not [17].

There are only limited prior studies on whether apheresis reduces cardiovascular events. These studies have been small, primarily nonrandomized trials. The LDL-Apheresis Atherosclerosis Regression Study (LAARS) looked at the change in plaque characteristics of patients undergoing apheresis compared to drug therapy over a period of two years [17]. In that period, 7 out of 21 patients on apheresis had a cardiac event compared with 3 out of 21 on medication only. While this study found that apheresis arrested the progression of atherosclerosis, it did not show that cardiac events were affected. The FH regression study found that LDL apheresis combined with simvastatin was more effective than colestipol plus simvastatin in reducing LDL cholesterol and lipoprotein (a) but was less effective at influencing coronary atherosclerosis [18]. Another study [19] found that, out of 18 patients, 3 had myocardial infarctions, 1 underwent a CABG, and 12 needed coronary angioplasties within two years of beginning a combination therapy of apheresis, statins, and probucol. Before beginning the combination therapy, 11 had experienced a MI, 5 had undergone a CABG, and 13 had undergone an angioplasty. The heparin-induced extracorporeal LDL precipitation (HELP) study [20] found that HELP is suitable for reducing LDL concentrations and may work to reduce the burden of atherosclerosis, as there were no myocardial infarctions and a low coronary intervention rate in patients who began apheresis.

Due to the expensive nature of apheresis, a randomized, controlled clinical trial is needed to truly gauge the effectiveness of apheresis in reducing the occurrence of cardiac events. If apheresis is not deemed effective or is minimally effective, as some of these studies suggest, other types of treatment, such as lomitapide, mipomersen, or PCSK9 inhibitors, should be pursued.

### 4.2. Survey Results

While satisfaction was generally high in our survey, patients specifically cited that this satisfaction was based on the results of apheresis and not on the process itself. Many patients complained about the invasive nature of apheresis, citing bruises from the procedure and the inconvenience of reporting for treatment every two weeks. Additionally, almost all patients were interested in learning more about alternative treatments, suggesting that they would prefer an alternative treatment which could match the results provided by apheresis.

### 4.3. Limitations

This study had several limitations. Since lipid apheresis is an advanced treatment for an uncommon genetic disease, the limited number of patients available to participate in the study led to a small sample size. The event rate reduction was not statistically significant but showed a strong trend toward cardiac event rate reduction before and after apheresis. By defining the observational period initial time point as the first documented EHR visit, we excluded 20 events before apheresis and 2 events after apheresis from the cardiac event rate calculation. The lipid-lowering effects of apheresis are best expressed as reductions in interval means. Although lipid apheresis was performed every two weeks, LDL values were not measured ever two weeks due to clinical practices. This inconsistency in measurement intervals prevents the use of more advanced measures to accurately track the effect apheresis has on LDL measurements. Also, this study was uncontrolled due to its retrospective nature. Finally, this study focused on active apheresis patients and therefore did not include patients who had stopped apheresis or were deceased.

## 5. Conclusion

Lipid apheresis can reliably reduce LDL, non-HDL cholesterol, triglyceride, and total cholesterol levels in FH patients. Our data suggest that lipid apheresis shows a strong, but not statistically significant, trend towards the reduction of cardiac events. Apheresis is a viable treatment for FH patients, especially those that are statin-intolerant, due to its lipid lowering nature and its apparent reduction of cardiac events. However, there is a need for alternative treatments which are less invasive and provide easier patient access.

## Figures and Tables

**Table 1:** Patient characteristics.

	*n* = 11
Age (years), mean (SD)	65.6 (9.3)
Male, (%)	8 (72.7)
BMI (kg/m^2^), mean (SD)	30.0 (5.3)
Current smoker, (%)	1 (9.1)
Former smoker, (%)	2 (18.2)
Diabetes, (%)	2 (18.2)
Hypertension, (%)	4 (36.4)
Blood pressure before apheresis	
Systolic (mmHg), mean (SD)	136.9 (16.3)
Diastolic (mmHg), mean (SD)	79.5 (6.7)
Blood pressure after apheresis	
Systolic (mmHg), mean (SD)	133.0 (13.3)
Diastolic (mmHg), mean (SD)	70.2 (8.1)
Statin intolerant, (%)	7 (63.6)
Cholesterol medication before apheresis	
Statin, (%)	5 (45.5)
Nonstatin, (%)	4 (36.6)
Cholesterol medication after apheresis	
Statin, (%)	4 (36.6)
Nonstatin, (%)	5 (45.5)

**Table 2:** Change in lipids with apheresis.

	Before	After	Change	*P* value
Total cholesterol (mg/dL), mean ± SD	286.8 ± 75.9	119.1 ± 25.9	−167.7 ± 60.4 (58.4%)	<0.001
LDL cholesterol (mg/dL), mean ± SD	192.09 ± 78.3	53.95 ± 22.1	−138.1 ± 58.7 (71.9%)	<0.001
HDL cholesterol (mg/dL), mean ± SD	47.2 ± 8.8	42.8 ± 8.3	−4.4 ± 5.4 (9.3%)	0.024
Triglycerides (mg/dL), mean ± SD	199.2 ± 105.4	97.6 ± 75.4	−101.5 ± 79.5 (51.0%)	<0.001
Non-HDL cholesterol (mg/dL), mean ± SD	239.6 ± 77.5	76.3 ± 22.8	−163.4 ± 61.9 (68.2%)	<0.001

**Table 3:** Questionnaire results.

Patient satisfaction (out of 5), mean (SD)	4.7 ± 0.7
Aware of FH	100.0%
Family tested for FH	70.0%
Family history of cardiac events	100.0%
Smoker∗	11.1%
Diabetes∗	11.1%
High blood pressure, treated or untreated∗	55.6%
Interested in alternative treatment	90.0%

^*^One patient did not complete the risk factor section and was excluded.

**Table 4:** Cardiac events.

	Before apheresis (*n* = 11)	After apheresis (*n* = 11)	Total
Event count			
Stent/PTCA	16	8	24
CABG	5	1	6
MI	13	0	13
Total cardiac events	**34**	**9**	**43**
Patient count			
Stent/PTCA	7 (63.6%)	4 (36.4%)	7 (63.6%)
CABG	4 (36.4%)	1 (9.1%)	4 (36.4%)
MI	5 (45.5%)	0 (0.0%)	5 (45.5%)
Total patient count with events	**8 (72.7%)**	**5 (45.5%)**	**8 (72.7%)**

			*P*-value

Cardiac event rate	0.23 (95% CI)	0.10 (95% CI)	0.064

## Appendix

### Questionnaire

(1) What date did you start apheresis (here or elsewhere)? _______________________.
(2) Overall, how satisfied are you with the apheresis program here at ANW hospital?
  (a) On a scale of 1–5 _______.
  1: not satisfied,
  5: extremely satisfied.
(3) Are you aware that you likely have Familial Hypercholesterolemia (FH), an inherited condition which runs strongly in families and is a genetic disorder? Y or N
(4) Has your immediate family been tested for Familial Hypercholesterolemia? Y or N
(5) Risk Factors:
  (a) Do you have family history of heart problems/cardiac events (did your mother or father, sister or brother have a heart attack, bypass, angioplasty at a young age)?
  (b) Do you smoke? Y or N
    (i) If yes, how often? ___________________.
  (c) Do you have diabetes? Y or N
  (d) How many minutes of moderate exercise do you get in a typical week? ____min.
  (e) Do you have high blood pressure (either treated or untreated)? Y or N
(6) Have you had any cardiac events (myocardial infarction, angioplasty, stents, CABG) before? Y or N
  (a) If so, how many cardiac events did you have before (_____) and after (_____) you started apheresis?
(7) Are you on any cholesterol-lowering drugs? Y or N
  (a) Please circle: Statins Welchol Zetia Niacin other: ________________.
  (b) Have you been intolerant to cholesterol lowering drugs? Y or N
  (c) If so which ones? ___________________________________________.
(8) Are you interested in learning more about FH or other treatments? Y or N.

## Abbreviations

ANW: Abbott Northwestern
CABG: Coronary artery bypass graft
EHR: Electronic health record
FDA: United States Food and Drug Administration
FH: Familial hypercholesterolemia
HDL: High density lipoprotein
HELP: Heparin-induced extracorporeal LDL precipitation
HMG-CoA: 3-Hydroxyl-3-methylglutaryl-coenzyme A
LAARS: LDL-Apheresis Atherosclerotic Regression Study
IRB: Institutional Review Board
LDL: Low density lipoprotein
MHI: Minneapolis Heart Institute
MI: Myocardial infarction
NLA: National Lipid Association
PCSK9: Proprotein convertase subtilisin kexin type 9
PTCA: Percutaneous transluminal coronary angioplasty.
